# Microbial Biofilms and Chronic Wounds

**DOI:** 10.3390/microorganisms5010009

**Published:** 2017-03-07

**Authors:** Amin Omar, J. Barry Wright, Gregory Schultz, Robert Burrell, Patricia Nadworny

**Affiliations:** 1 Innovotech Inc., Suite 101, 2011 94 Street, Edmonton, Alberta T6N 1H1, Canada; amin.omar@innovotech.ca (A.O.); patricia.nadworny@innovotech.ca (P.N.); 2 Harkynn Consulting, P.O. Box 104, Albertville, Saskatchewan S0J 0A0, Canada; jbarry.wright@gmail.com; 3 Department of Obstetrics and Gynecology, Institute for Wound Research, University of Florida, 1600 South West Archer Road, Room M337F, Gainesville, FL 32610-0294, USA; schultzg@ufl.edu; 4 Department of Biomedical Engineering, Faculties of Engineering and Medicine & Dentistry, 1101 Research Transition Facility, University of Alberta, Edmonton, Alberta T6G 2G6, Canada

**Keywords:** quorum sensing, antibiotic tolerance, antibiotic resistance, exopolymer, inflammation, wound healing, MBEC (minimum biofilm eradication concentration)

## Abstract

Background is provided on biofilms, including their formation, tolerance mechanisms, structure, and morphology within the context of chronic wounds. The features of biofilms in chronic wounds are discussed in detail, as is the impact of biofilm on wound chronicity. Difficulties associated with the use of standard susceptibility tests (minimum inhibitory concentrations or MICs) to determine appropriate treatment regimens for, or develop new treatments for use in, chronic wounds are discussed, with alternate test methods specific to biofilms being recommended. Animal models appropriate for evaluating biofilm treatments are also described. Current and potential future therapies for treatment of biofilm-containing chronic wounds, including probiotic therapy, virulence attenuation, biofilm phenotype expression attenuation, immune response suppression, and aggressive debridement combined with antimicrobial dressings, are described.

## 1. Introduction—Microbial Biofilms

Despite the focus of microbiological research on planktonic (single organisms, suspended/free floating) populations of bacteria, it has become apparent that bacteria exist predominately within biofilms in natural and clinical settings [1]. Research over the past few decades has now clearly established that 99.9% of all known microorganisms in natural settings are attached to surfaces, due to the nutritional and protective benefits associated with life in an adherent population [2]. Once bacteria attach to a surface, they produce complex exopolymers—containing polysaccharides, proteins, and nucleic acids [3,4,5]—which help both to preserve their attachment to the surface and to maintain bacterial cells in close proximity to each other. As the number of bacteria present in a specific area increases, this adherent population is referred to as a biofilm.

## 2. Biofilm Formation

Biofilms are three-dimensional mosaic consortia of microbes, which accumulate and organize at surfaces within an extracellular polymer, or glycocalyx, with interspersed water channels [6,7]. The vast majority of the biofilm’s volume, 80%–85%, is comprised of the exopolymer, with the remaining 15%–20% consisting of microorganisms [2]. The high content of water in a biofilm contributed to the challenge of its original observation, as the otherwise unsupported exopolymer collapses onto the bacterial surface during dehydration for electron microscopy [2].

**Figure 1:**
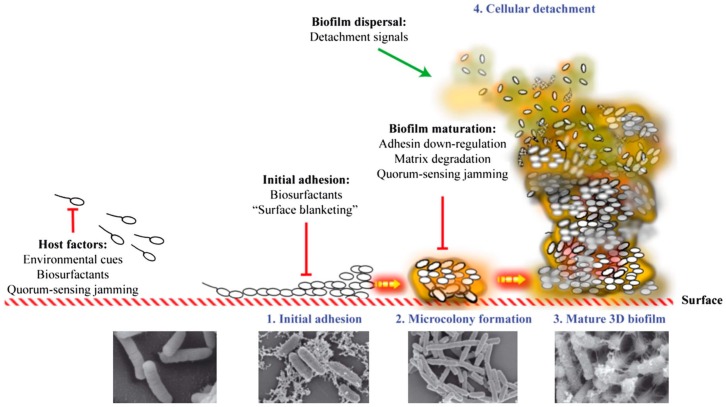
Stages in the biofilm formation process, including scanning electron microscopy imaging of each stage. Reproduced from [12], by permission of Oxford University Press and FEMS.

Bacterial biofilm formation begins when a planktonic bacterium finds its way to an exposed, conditioning, film-coated surface through Brownian or flagellar motion (see Figure 1). It then overcomes the electrostatic repulsive forces between the substratum and the bacterial cell surface, and makes initial attachment to that surface [8]. Different types of biofilms can then form, depending on the environment, including pellicles that form at air–liquid interfaces and solid surface-associated submerged biofilms [9]. Once attachment to a surface has occurred, a microcolony consisting of primary colonizers rapidly develops [10]. At this point, bacteria are encased in a protective matrix and are beginning to express the biofilm phenotype (see Figure 1). This includes becoming more recalcitrant to antimicrobial treatment and host immunity. The bacterial cells within the nascent microcolony release quorum sensing molecules (small molecular weight molecules), which eventually reach a critical concentration as the adherent population increases in size (see Figure 1). At this critical level, the quorum sensing molecules trigger a changed expression of specific genes, helping the bacterial community to form a mature biofilm. Once the biofilm establishes and matures on a surface, it releases planktonic cells that migrate back into the bulk fluid phase until they find a new location to colonize (see Figure 1) [11].

## 3. Quorum Sensing

Quorum sensing is a key process involved in the formation of biofilms, and in the expression of biofilm-specific properties. Quorum sensing molecules, called autoinducers (AIs), have been identified in many species of bacteria, with a variety of molecules playing a quorum sensing role, depending on the particular species. Many different classes of AIs have been described. The most intensely studied are the *N*-acylhomoserine lactones (AHLs) produced by Gram-negative bacteria [13]. In addition, Gram-positive bacteria appear generally to produce small peptides as well as a class of molecules, called AI-2, for which the structures are largely unknown [14,15]. Regardless of the type of molecule involved, these AIs are small molecules that are produced at a basal rate in bacterial cells. Some of these AIs are freely diffusible across bacterial membranes, in which case, their intracellular concentration approximates that in their surrounding environment. As the number of bacteria increases within a localized area (microcolony), the effective concentration of the AI is elevated. Once the intracellular concentration of AI reaches a critical level, the AIs induce a number of changes in gene expression. This results in the properties typical of a biofilm, among other changes. The impact of some of these molecules on the morphology of the biofilms formed is discussed in more detail in Section 5 below.

Interestingly, two of the bacterial species in which quorum sensing has been most intensely studied, *Staphylococcus aureus* and *Pseudomonas aeruginosa*, are also commonly associated with chronic wounds. The involvement of these organisms and biofilms in chronic wounds will be explored more fully below. *S. aureus*, like other Gram-positive bacteria, utilizes a peptide quorum sensing system that has served as a model of peptide-based quorum sensing [16]. In this species, quorum sensing is mediated by autoinducing peptides (AIPs) encoded by the *agr* locus. This same locus also controls a number of virulence factors, including biofilm formation [17,18].

Similarly, the quorum sensing mechanisms of *P. aeruginosa* have also been extensively studied. Many of the virulence factors of this chronic wound colonizer have also been observed to be under the control of the quorum sensing pathways, including secreted virulence factors (e.g., proteases), cell-attached factors (e.g., lipopolysaccharide), and biofilm formation [19]. Quorum sensing in this organism has an effect very early in the development of biofilms, with quorum sensing mutants being unable to form structurally normal biofilms [20].

Additional evidence exists for the involvement of quorum sensing in biofilm processes in vivo, which includes the isolation of pseudomonal AIs at significant concentrations in cystic fibrosis patients colonized with *P. aeruginosa* [21]. However, numerous quorum sensing mutants are also isolated from the sputa of cystic fibrosis patients. This suggests that the quorum sensing must exact a cost upon the population such that it is not necessary for all of the members of the population to have a functional quorum sensing system [22,23]. Despite these observations, recent work has demonstrated that the production of quorum sensing controlled extracellular factors is more efficient at higher cell densities and provides a fitness benefit to the population [24]. It also appears that quorum-sensing molecules exert a direct influence on host cells, altering the host’s cellular functions, including activities such as inhibiting lymphocyte proliferation [25].

## 4. Biofilm Resistance

Biofilm communities are highly beneficial to many aspects of human life, including the provision of colonization resistance to the large intestine [26], degradation of organic compounds and environmental pollutants [27], global nutrient cycling [28], and improvement of water quality [6]. However, these metabolically integrated multicellular communities are largely regarded as problematic in both industrial and clinical settings. This is because biofilms are extremely recalcitrant to elimination by antimicrobial agents and the host’s immune response. Biofilms are generally reported to be far less susceptible to antimicrobial treatments than their planktonic counterparts, with 100–1000× decreases in susceptibility, or more, frequently demonstrated [29,30,31].

There is in vivo and in vitro evidence that the exopolymer protects chronic wound biofilms from the inflammatory processes that are key to wound healing [32]. The exopolymer has been suggested to block complement activation [33], depress the lymphoproliferative response [34], and prevent detection of opsonins on bacterial cell walls by phagocytes. Findings have also shown that the exopolymer limits the ability of leukocytes to penetrate the biofilm [32], hampers their movement through biofilms, attenuates their ability to degranulate and produce reactive oxygen species (ROS), and prevents the phagocytosis of bacteria [31,35]. Exposing bacterial biofilms to sub-inhibitory antibiotic concentrations, or to the wrong antibiotics, may induce mucoid phenotypes, which generate thicker biofilms with additional matrix components [36,37,38].

Biofilm-related infections are notoriously hard to eradicate and have been the subject of intense scientific research over the past 30 years [6]. Examples of biofilm-associated infections include the colonization of implanted medical devices [39,40] such as central venous catheters, joint prostheses, urinary catheters, pacemakers, and mechanical heart valves; dental caries; lung infections in cystic fibrosis patients [41]; and chronic wounds [40]. The majority of human infections (60%–80%) are biofilm associated [42,43].

Biofilm-associated cutaneous diseases include burns, pressure ulcers, surgical site infections, and diabetic foot ulcers. Their annual incidence is 1.96 million cases in the United States, causing an estimated 268,000 deaths, and an estimated annual direct cost of $18 billion [44].

Various mechanisms have been proposed to explain the tolerance of biofilms to aggressive treatment therapies. Theories include:

- The physiological heterogeneity of the biofilm consortia [45]
- The presence of persister cells that allow for repopulation of the biofilm after treatments [46,47,48]
- Low metabolic rates of biofilm-associated bacteria, which impact the mechanism(s) of action of commonly used antibiotics [2,48,49]
- Overexpression of efflux pump open reading frames [50]
- Drug diffusion limitations caused by the exopolymer [29]
- The predominance of drug-resistant genes, which can easily be transferred to other organisms within a biofilm [51]

No single mechanism serves to adequately explain the long-term chronicity of these communities, but rather a combination of mechanisms appears to be involved.

It is important to note here that the term “resistance” describes a permanent alteration of a microorganism’s genes that is passed on during proliferation and enables planktonic microorganisms to survive exposure to antimicrobial agents that normally kill nonresistant strains of the species, meaning that resistance is not dependent on the physical growth conditions of the microorganism. “Tolerance”, on the other hand, describes the transient ability of microorganisms to survive exposure to agents that normally will kill the planktonic form of the species. Tolerance is usually dependent on the physical status and/or conditions immediately surrounding the microorganism, such as a biofilm matrix and metabolically dormant persister cells.

## 5. Biofilm Structure and Morphology

Originally, biofilms were thought to be amorphous aggregations of microorganisms. This view was supported by evidence that biofilms from a wide variety of environments are similar in structure. Evidence was then found that both genetic (cell signaling and differences in exopolymeric substance production) and environmental (nutrient and fluid flow) factors were responsible for the biofilm structure and morphology [52,53]. There is now clear evidence that biofilm structure is more organized than previously thought, with the discovery of nanowires and honeycombs [54].

Studies have demonstrated that *P. aeruginosa* colony geometries are optimized with respect to growth efficiency [55,56]. Using colony morphology assays and mathematical modeling, studies have shown that *P. aeruginosa* forms tall ridges or wrinkles (also referred to as colony rugosity) to enhance access to oxygen in response to reduced cellular redox status [55,56]. These wrinkles reach a static width, which correlates to the concentration of oxygen in the environment, while continuing to grow taller, indicating that electron acceptor rather than nutrient supply is the primary limitation causing these features to develop [55,56]. Production of endogenous redox-active antibiotics called phenazines allows for formations with lower surface areas (i.e., wider/fewer wrinkles) [55]. This is because phenazines act as electron acceptors in anoxic regions of biofilms and shuttle electrons to well-aerated regions, allowing cells to balance their internal redox state, thus extending the depth of respiration/habitability within biofilms [55,56,57,58,59]. Phenazines do not appear to impact the ability of *P. aeruginosa* to attach to surfaces, but do impact swarming motility (possibly by regulating flagellar function) and biofilm surface-to-volume ratios, as described above; different phenazines can have different impacts on colony structure, and phenazines can have different impacts on different bacterial species [60]. Exogenous nitrate (an alternate electron acceptor utilized by *P. aeruginosa*) has a similar effect on biofilm morphology to that demonstrated by phenazines [55,56]. Availability of oxygen and/or alternate electron acceptors also impacts colony base depth and total colony surface area (i.e., spreading plus wrinkles), with a growth optimization occurring between sufficient oxygenation of cells and the total number of cells contained within an area [55]. Other cellular behaviors that impact biofilm structures include chemotaxis, extracellular matrix (ECM) production, chemical signaling, and selective cellular death as an induction for mechanical buckling [55,61,62,63]. Impaired respiration, including via low iron levels resulting in defects in the assembly of the cytochromes in the respiratory apparatus, has also been shown to act as a signal for triggering matrix production in *Bacillus subtilis* [64]. This was via kinases KinB (interactions with respiratory apparatus) and KinA (sensing drops in the nicotinamide adenine dinucleotide (NAD^+^/NADH) ratio), again with resultant biofilm formation and wrinkling allowing for increased accessibility to oxygen via increased surface area [64].

A recent study has also demonstrated that *B. subtilis* and *Mycobacterium smegmatis* maintain biofilm structures (e.g., thicker and/or wrinklier films) via the production of calcite minerals [65]. This production is triggered by a rise in carbon dioxide levels, promoted by an intrinsic alkaline environment in the colony, and facilitated/directed by extracellular matrix components [65]. These mineral scaffolds—which provide physical stability, resistance to environmental insults (including antibacterial agents), and increased overall fitness in the biofilm (including precipitation of toxic carbon dioxide)—may play a cardinal and conserved role in bacterial multicellular communities [65].

The recognition of pathogenic bacteria—in periodontitis, in the lungs of patients with cystic fibrosis, or in chronic wounds—existing as members of highly organized communities means that strategies can now be developed for disrupting these communities. Although biofilms are now recognized as very complex communities, the single organism and its role in developing a biofilm community should not be ignored.

This leads to the question: Can some aspect of the biofilm phenotype be expressed by a single cell? Experimental evidence suggests this may occur and, if it does, this would have a major impact on the antimicrobial treatment of wounds [66].

Chan demonstrated that *P. aeruginosa* sensitivity to antibiotics increases as the viscosity declines to that of water [67]. *P. aeruginosa* grown in the presence of gentamicin at high viscosity (15% polyvinylpyrrolidone (PVP)) had a minimum bactericidal concentration (MBC) that was 6 times higher (*p* = 0.01) than that of cells grown in a medium with the viscosity of water. Similarly, this organism was 11 times (*p* = 0.0001) less responsive to piperacillin at a high viscosity (12.5% PVP) than it was at low viscosity. When *P. aeruginosa* was grown for a period of time at a high viscosity, and the viscocity was then changed through dilution (1:100) followed by 3 h of incubation, the MBC values returned to values similar to those of cells grown at the viscosity of water [67].

Further, the growth rate and, in particular, the final cell numbers generated also increased in conjunction with increased growth media viscosity for both *P. aeruginosa* and *Candida albicans* [67]. *P. aeruginosa* and *C. albicans* produced 3 and 10 times more cells, respectively, at higher viscosities than did the controls, which were grown at the viscosity of water [67].

These organisms thus appear to behave like a biofilm in high-viscosity environments, and like planktonic organisms as the viscosity decreases. This suggests that decreased metabolic activity is not directly related to tolerance and that the biofilm phenotype can be expressed without attachment to a substratum. These observations could have a profound effect on wound care. A highly exudative wound is typically infected and highly inflamed. The presence of inflammation and exudate suggests the presence of a large, nearby supply of blood, yet systemic antibiotics seldom work well against bacteria in chronic wounds. This was usually blamed on poor vascularization, but is perhaps more related to the presence of microorganisms expressing a biofilm phenotype. In moderately to highly exudative wounds, evaporation of water results in a viscosity increase in the residual fluid through an increase in protein concentration. Organisms that are resident in this higher viscosity exudate may be capable of expressing the biofilm characteristic of reduced antibiotic sensitivity, making them difficult to control. This may also explain the problems in maintaining antimicrobial control in the lungs of cystic fibrosis patients.

## 6. Biofilms in Chronic Wounds

Bacterial biofilms have now been clearly identified in chronic wounds. Early evidence of bacterial biofilms existing in wounds was derived from experimentally induced chronic wounds in animals and was subsequently demonstrated in clinical wounds [68,69]. Considerable heterogeneity exists in the bacterial colonization of chronic wounds, with pathogenic bacteria becoming the dominant microflora at the expense of commensal species [35,70].

The type and relative numbers of bacteria present differ significantly from wound to wound [2,49]. One study demonstrated up to 17 genera of bacteria (aerobic and anaerobic) per wound [49]. Another indicated that 12–20 different species of microorganisms per wound was typical, with 60 different types not being uncommon [2]. A number of studies have suggested that the more abundant the microflora of the intact skin, the greater the protection from the spread of infection or the accumulation of both opportunistic and strictly pathogenic species [49,71,72]. However, the introduction of certain pathogenic species (notably *S. aureus* and *P. aeruginosa*) into the environment can cause replacement of harmless skin commensal organisms, leading to a shift to an infected state. This suggests a possible role for probiotic therapy in chronic wound care that could reverse the microbial ecology back to a healthy state [35,70,73]. A recent example of this concept is the successful use of fecal transplantation in patients with severe, recurrent gastroenteritis caused by overgrowth of *Clostridium difficile*. In these patients, inoculation of the intestines and colon via an enema with “healthy commensal” bacteria, derived from the feces of healthy individuals, allowed for restoration of a normal, commensal bacteria population, effectively reversing the gastroenteritis [74].

Oxygen limitations, deeper in the biofilm, promote the proliferation of anaerobes in chronic wounds [54,70]. Thus, rather than focusing on the bacterial load of a wound, consideration of the species present and their interactions within the wound is also important. This includes determining whether the species coexist to proliferate or compete [2]. Frequently repeated patterns of co-aggregating species have been observed that exhibit the ability to synergize, producing chronic biofilm wound infections. These are referred to as “functional equivalent pathogroups”, of which there may be hundreds [47].

As well as containing multiple species, biofilms contain cells at all stages of the growth cycle. Deep within the biofilm, there are microorganisms with reduced microbial growth rates that are related to a general stress response, which protect the bacteria from the effects of pH changes, chemical agent concentration, osmolality, and the effects of chemicals that require active bacterial growth to be effective [48]. Furthermore, biofilms contain many concentration gradients, creating microenvironments that have a negative effect on antibiotics and antiseptics, while, in some cases, promoting the growth of particular microbial species. These microenvironments can include aerobic and anaerobic microenvironments [48]. Although some authors have suggested that the majority of biofilm cells appear to be killed by antibiotics, rapid regrowth of biofilm occurs in chronic wounds after treatment. This suggests the presence of persister cells within these biofilms [75,76,77]. Persister cells are estimated to constitute 0.1%–10% of a biofilm, and also exist in planktonic cultures [78]. They are thought to be phenotypic variants that demonstrate antibiotic tolerance via phenotypic changes, such as being metabolically quiescent and/or turning off antibiotic targets [48].

The lack of antimicrobial effectiveness may be related to reduced or incomplete penetration of antimicrobials into the biofilm. The net negative charge of the exopolymer could sequester positively charged compounds and/or repulse negatively charged compounds, preventing contact with the microorganisms within the biofilm [48]. Some organisms also release molecules that sequester antibiotics [48]. Overall, this makes the treatment of chronic, biofilm-associated infections much more challenging than treatment of acute, planktonic infections.

Unfortunately, many of the treatments currently available were designed to treat acute infections, which, unlike chronic infections, tend to appear quickly and run their course over a short period of time. Planktonic bacteria typically respond to antibiotics and are easily exterminated by a healthy immune system [79]. In contrast, chronic wounds are normally characterized by a tenacious and excessive inflammatory response when compared with acute wounds [40] and are less susceptible to antibiotics.

Mature biofilms develop in chronic wounds as early as within 10 hours, and persist indefinitely while the wound remains open [80,81]. In addition, a clinical study found that although surgical debridement of chronic wounds effectively removed biofilm communities from the wound beds, biofilms began to reemerge 2 days after the initial debridement, and high numbers of bacteria in the mature biofilms were identified 3 days after debridement [82]. This indicates there is a window of opportunity, after debridement, during which the planktonic bacteria recolonizing the wound bed are susceptible to treatments that can effectively kill them, and prevent the reformation of biofilm communities.

Biofilms are microscopically identifiable in up to 60% of chronic wounds, but in only 6% of acute wounds [83]. Studies have also linked the presence of suspected biofilms in chronic wounds (determined visually) with acute wound infection, chronic bone infection, moisture imbalance, and underlying arterial flow impairment [84]. Chronic biofilm infections are persistent and very hard to eradicate. They respond incompletely to antibiotics, prescribed based on minimum inhibitory concentration (MIC) test results, and often recur once the course of antibiotics is finished.

Even so, the biofilm that is formed in chronic wounds may differ from that observed in other types of infections. In fact, in many chronic wounds, the existence of a true biofilm may be difficult to demonstrate. However, the formation of microcolonies (i.e., relatively small numbers of cells in close proximity) within the wound environment is generally evident. Given that some species of bacteria, such as *P. aeruginosa*, have been shown to exhibit the effects of increased levels of AIs at the microcolony stage, and that some AIs have the ability to have a direct impact on human cells, the presence of these microcolonies may be sufficient to promote the chronicity of wounds. For example, if microcolonies are sufficiently large to prevent their engulfment by phagocytic cells, the resultant frustrated phagocytosis may cause degranulation of some phagocytic cells. This results in an excessive immune response that may alter the balance of a variety of factors in the wound, for example, the matrix metalloproteases that have been postulated to inhibit normal wound closure. When such an immune response can be attenuated, the wound may heal normally, and rapidly [85,86].

## 7. Identification of Drug Resistance/Tolerance, Susceptibility, and Treatments

Despite recommendations for the use of standard susceptibility testing [87], the clinical utility of standard MIC susceptibility testing has been called into question [88]. MICs measure the action of antibiotics against planktonic organisms and serve as an important reference in the treatment of many acute infections. However, the application of MICs to the treatment of chronic and device-related infections that involve bacterial biofilms is often ineffective [43].

Research has examined the susceptibility of a large number of cystic fibrosis clinical isolates of *P. aeruginosa* growing as biofilms, and compared these results with standard MIC determinations [89]. The results identified a correlation between biofilm antimicrobial tolerance and airflow obstruction. The patients who received antibiotics that *P. aeruginosa* biofilms were sensitive to, based on a biofilm susceptibility in vitro diagnostic test, demonstrated significant decreases in sputum bacterial density and length of hospital stay [89]. They also demonstrated significant improvements in clinical outcomes, relative to patients who received antibiotics based on MIC testing [89].

Evidence of the in vitro anti-biofilm activity of antibiotics tested against *P. aeruginosa* strains grown as biofilms, and of different susceptibility patterns determined by the two methods (biofilm vs. standard MIC) supports the feasibility of adapting biofilm susceptibility testing methods to the clinical microbiology laboratory [90]. This opens the way to selecting more effective antibiotic combinations for chronic infections than are selected by methods in current use [90]. An in vitro model has been tested on burn wound organisms; the authors concluded that the assay was practical, reproducible, and useful for the selection of antibiotics to treat burn wound biofilms [91]. While the test is simple to run, the biofilms are only 24 h old when tested and, based upon what is known about the biofilm maturation process, the test may need further refinement [91]. A unique ASTM-approved in vitro device (MBEC™, Innovotech, Edmonton, AB, Canada; ASTM E2799-12 [92]), has been developed to address these issues and may be used for testing both planktonic and biofilm susceptibility profiles of many Gram-negative and Gram-positive non-fastidious bacterial isolates at serum breakpoint levels [90,92].

Another difficulty in developing strategies to treat biofilm infections is the lack of in vivo model systems to evaluate treatment regimens. The tests that are currently available include murine models [93] and porcine models [68,69,85]. The murine model is disadvantaged by a healing process that occurs by contracture, making the results not readily applicable to clinical situations. Furthermore, the biofilms for the murine model are developed in vitro and applied to the wound, which may affect the host response. Healing in a porcine model is by re-epithelialization and is well-established as being similar to human wound healing [94]. In the porcine model, wounds are directly inoculated with a mix of aerobic and anaerobic organisms, and the biofilm phenotype forms in situ. In both models, the host effects on the resolution of the biofilm infection is unclear. Regardless, models that demonstrate results that are the most immediately reflective of the human situation are the most beneficial for the promulgation of useful clinical treatments.

## 8. Research on New Treatment Strategies

There are increasing numbers of bacteria that are genetically resistant to antibiotics. Additionally, even bacteria that are not genetically resistant to antibiotics may be tolerant to antibiotics by virtue of their mode of growth. The problem of pan-resistant bacteria and the need for novel ways to address this problem is growing increasingly urgent [95]. Despite this, few pharmaceutical companies are exploring new antibiotic therapeutics [96]. Part of the reason for this is the difficulty (and expense) of discovering new drugs that can be administered to patients at concentrations sufficiently high to be effective against the bacteria, without harming the patient. This difficulty, combined with the (typically) short-term duration of antibiotic therapy tends to make this field financially unattractive. Therefore, virulence attenuation approaches are being considered that involve drugs that do not actually kill the bacteria. Rather, these compounds interfere with the ability of the bacteria to produce virulence factors, such as the factors produced during growth as biofilms, which promote resistance to existing drugs. This approach is particularly attractive because it theoretically applies less selective pressure on the bacterial community, thereby minimizing the development of novel antibiotic resistance mechanisms [97].

Unfortunately, recent research in this area, focusing on *P. aeruginosa*, has provided a large dose of realism. In experiments looking at the development of resistance to compounds that inhibit quorum sensing, the bacteria appear to be able to develop resistance to these molecules, as well, through the development of highly efficient efflux pumps [98]. Thus, reverting to strategies that maintain the bacterial population under some level of control and that simultaneously discourage over-exuberant immunological responses may be necessary to allow the host to resolve the infection. For the present, in chronic wounds, such strategies may involve the use of some antimicrobial dressings combined with techniques such as aggressive debridement.

A number of potential anti-biofilm treatments currently under investigation are based on the fact that biofilms eventually disassemble as a means for surviving cells to leave the biofilm and be dispersed, particularly as resources become limited and waste products or other toxins accumulate [99,100,101]. Biofilm disassembly often involves the induction of enzymes that destroy components of the matrix and, thus, liberate the biofilm-associated cells; therefore, enzymatic treatment may be one approach to biofilm eradication [99,102]. As well, recombinant phages have been developed that attack biofilm cells and produce a matrix-degrading enzyme [99,103]. In addition, given that biofilm formation requires cell–cell signaling, some investigators feel that small molecules that would interfere with these signaling pathways might prove useful for biofilm eradication [99]. Small molecules can also naturally trigger and mediate the disassembly of biofilms via at least four different mechanisms, including:

1. Signals and cues:
  - AIP-1 (Gram-positive bacteria)—controls the *agr* system based on quorum sensing such that disassembly of the biofilm is coupled to increased density or decreased nutrient availability, causing release of proteases and pore-forming toxins, and increased expression of virulence factors [100,104,105,106]
  - Diffusible signal factor (DSF—Gram-negative bacteria)—orchestrates biofilm disassembly based on oxygen availability and density, triggering autophosphorylation and enhanced virulence gene expression [100,107]
  - *cis*-decenoic acid (Gram-negative bacteria)—appears to initiate disassembly through transcriptional regulation [100,108]
  - Siderophores such as pyoverdin (iron chelator)—absence of sufficient iron uptake promotes biofilm disassembly [100,109]
2. Cell envelope-modifying molecules:
  - d-tyrosine, d-leucine—interfere with anchoring of protein amyloid fibers that assist in holding biofilms together [100,110,111]
  - Zaragozic acid—perturbs architecture of microdomains in cell membranes, causing mislocalizing of signaling proteins and general perturbation of bacterial lipid rafts [100,112,113]
3. Anti-matrix molecules:
  - Norspermidine (and related)—targets polysaccharide component of extracellular polymeric substance (EPS) [100]
  - AA-861, parthenolide—target specific protein component (TasA) of ECM [100]
  - Rhamnolipid and other bacterial surfactants—reduce surface-bacteria interactions [100]
4. Molecules that promote cell death:
  - Nitric oxide—induces programmed cell death and dispersal of bacterial biofilms [100,114]

Challenges with applying these potential anti-biofilm treatments include that the signaling molecules required for activation of disassembly can be species and even strain dependent; some quorum sensing pathways may only be important in initial biofilm formation and, therefore, may not be ideal for disassembly of pre-established biofilms [100,115]. Further, many small molecules, particularly anti-matrix molecules, have dual roles, causing biofilm formation, maturation, maintenance, or disassembly under different conditions [100].

## 9. Summary

Due to their continued persistence, biofilm infections cause more damage and greater inflammatory responses than the corresponding infections caused by planktonic bacteria. Therefore, the use of suitable antimicrobial agents to treat such infections is critical. It is time to take a step forward and adapt the new approaches to in vitro biofilm susceptibility testing in such a way as to reinvent the methods by which clinical and industrial microbiology testing and treatment are performed. Consider, for example, this quote from J. William Costerton:

“Those of us in the medical business must think very hard if we are to out-maneuver this very old and very successful bacterial life form, and perhaps learn to speak their language, and even enlist them in our never-ending fight against disease” [116].
